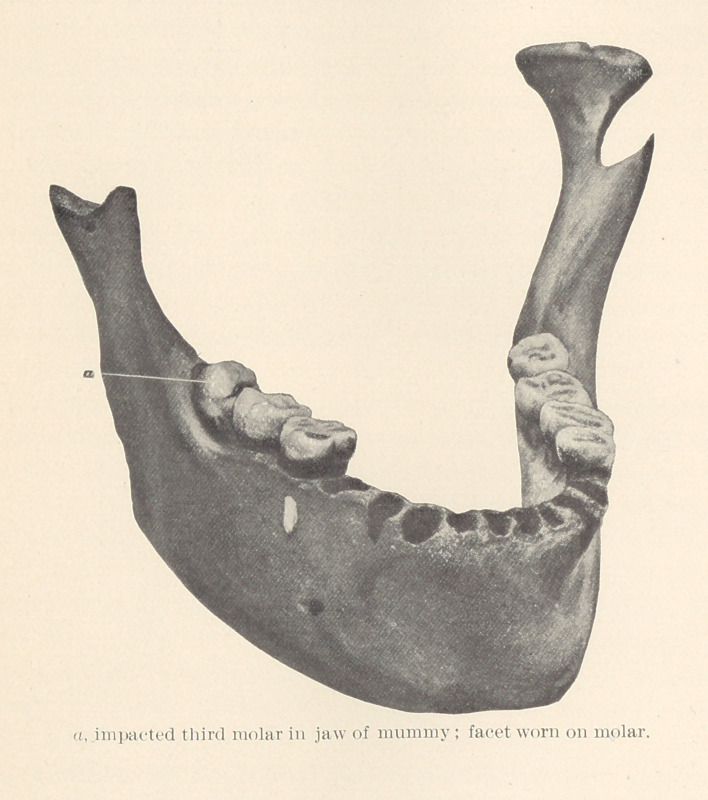# The Non-Specificity of Micro-Organisms in Dental Decay

**Published:** 1901-05

**Authors:** William H. Potter


### Reviews of Dental Literature.

THE NON-SPECIFICITY OF MICRO-ORGANISMS IN DENTAL DECAY.—J. Choquet sent a paper to the meeting of the Odontological Society of Great Britain on January 28, 1901, on the above subject, which was read in his absence. This very important matter he, however, failed to make clear, but regards this paper as prefatory to others of more decided character.

He says, "It is our opinion that one of the most interesting points is to prove that, in contradiction to the opinion of a few authors, there is no specific micro-organism of dental decay. That is the point which I will try to study in this paper. The work I have the honor to present to the Odontological Society of Great Britain is the prelude of others in which I will show good proof that the greatest number of micro-organisms found in saliva or elsewhere can produce the histo-pathological disorders which we are accustomed to see. I hope this first paper will shake the theory of specificity. I have obtained very good results proving the truth of my theory, but these results I will give in a few months."

His conclusions are—

"1. There is no specific microbe of dental decay.

"2. Dental decay is produced by microbic associations. The microbic elements may be pathogenic or not.

"3. The starting-point of dental decay is the saliva and its contents."

The discussion which followed the reading of M. Choquet's paper is not only interesting but it assumes a degree of authority, coming, as the remarks did, from men of world-wide reputation in this particular line of scientific investigation.

DISCUSSION.

*Mr. J. H. Mummery.*—I do not think that either Dr. Williams or I suggested that *leptothrix racemosa,* as such, penetrated the tubes of the dentine, or was concerned in caries of the teeth, whatever part its supposed derivatives may take in caries. We stated that an organism such as the higher phase of *leptothrix racemosa* existing in immense quantities in the mouth must have some important significance. With regard to leptothrix in the tubes, I certainly have seen many specimens of carious dentine showing coiled thread-forms in the tubules, but of course it is open to question if such thread-forms should be classed as leptothrix, leptothrix being a term which has been much misused and applied to many different forms. I think it has been claimed by most bacteriologists who have given their attention to the subject that there are many acid-forming organisms in the mouth, and that several or all of these may cause dental caries. M. Choquet speaks of further papers on the subject, and we shall look forward with interest to further evidence in proof of his views, his present paper hardly carrying us very far in the direction of evidence. We much regret M. Choquet's unavoidable absence this evening, as in the discussion he would no doubt have enlarged on several of his points.

Mr. Kenneth Goadby agreed with what Mr. Mummery had said, and was extremely sorry M. Choquet was not present to give the proofs of his theories. He gave no proofs, but simply a mass of conjectures, which were more or less common to all workers on dental caries. He was somewhat surprised at the suggestion that Dr. Choquet's conception was an original one, because in the first place Miller pointed out that the first stage of caries was an acid production by the organisms on the surface of the teeth, and that probably the resulting process was one due to putrefaction. Then in a paper read before the Society in 1898 he (Mr. Goadby) went at some length into the question, and stated definitely that the organisms in caries might be classed under three heads. The third heading was perhaps more or less a provisional one, but the other classes to which he suggested the organisms of caries should be referred were those of acid-producing organisms and those of liquefying organisms. He went at some little detail also into the liquefaction of dentine by organisms which were capable of liquefying blood-serum. Of course, he did not mean that because an organism liquefied blood-serum therefore it would liquefy dentine, but it was a somewhat curious fact that those organisms which liquefied blood-serum liquefied also decalcified dentine.

Dr. Choquet suggested that the decalcification of dentine and the liquefaction of it were two processes to be considered from different physiological points of view of the organism, and that in one case it was a direct process, and in the other, the digestive, an indirect process. He did not quite know why Dr. Choquet divided those two processes. Both of them were nothing more nor less than the result of the activity of special organisms. It was the common property of dental bacteriologists and bacteriologists generally that any destruction of tooth-substance could not be anything but one related to the process of putrefaction, that was, a non-specific process. Dr. Leon Williams had stated that certain organisms he had observed produced acid. He (Mr. Goadby) criticised him at the time because Dr. Williams said he had a complete demonstration, when only a few organisms were described. Since that time he had had much pleasure in confirming much of Dr. Williams's work, and had obtained many of the organisms from the surface of the teeth which were capable of producing acids under given conditions, not always from the same carbohydrate, not always under the same conditions, and not alone, but in conjunction with other species. Some would produce acid from lactose when grown in the presence of a second one, but would not produce acid in the lactose solution by itself. He had always considered that the putrefaction to which caries was clearly allied a symbiotic process producing as final products carbon dioxide and hydrogen, so that he thought the specificity of organisms in dental caries was a question scarcely necessary of discussion. The most happy remark of Dr. Choquet was his allusion to the fact that the organisms described by observers differed in all cases. If Dr. Choquet referred to one of his (Mr. Goadby's) papers, he would find that he had followed Dr. Miller's work as far as possible. He had been able to confirm in one or two cases the organisms Miller described, and his own description appended had been of a sufficiently precise nature for a bacteriologist, in the modern acceptance of the term, to recognize the organism. But the difficulty was that the majority of the processes that had been used in the description of bacteria were processes that were at present considered inadequate. He cited as a parallel case Lister's early work. Lister discovered an organism which he named *B. lactis,* and described the method of obtaining it in pure culture; the organism in question was thought to cause many of the conditions to which wounds were then liable. Later observers however, among them Hueppe, found that Lister's *B. lactis* was not a single species, but a mixture of about five distinct organisms, which were eventually isolated in pure cultivation. The principal part of dental bacteriology coincided with the period at which the work was done under the conditions he had just mentioned, and it had been extremely difficult to follow up from the writings of other observers the reactions they had given. He thought the question of observers not entirely confirming one another was due to their non-adoption of standard methods. He hoped to be able to recognize Dr. Choquet's organisms, although the descriptions given were insufficient.

Dr. Leon Williams said that Mr. Goadby had pretty well covered the ground, and left him very little to say. Four years ago, or even no longer than two years ago, there would, perhaps, have been more excuse for the subject-matter of the paper, because at that time belief in the specificity of the micro-organism of decay was pretty general. It led him a little less than two years ago, while he occupied the editorial chair of the *Dentist,* to point out clearly that there was no specific organism of decay, and that the relation of micro-organisms to dental decay was totally different from that in the case of specific diseases produced by micro-organisms. It was possible that in the circle in which Dr. Choquet moved the old belief still was held to a considerable extent, and that might be his justification for the position he had taken up in the present paper. The whole question of the specificity of micro-organisms was one which, at least, was open to discussion. The specificity was not so circumscribed as had been thought, but it was hardly necessary to go into that that evening. It all circled round one statement, that no fact could stand alone. The question of the specificity of micro-organisms was a question which involved the whole history of bacteriology. He should like to confirm what Mr. Mummery had said with reference to the relation between *leptothrix racemosa* and decay. He knew nothing which would support any statement that it was directly concerned in decay. It was always a great misfortune when the writer of a paper was not able to read it, as one felt a hesitancy in saying critical things when an author was absent.

Dr. Eyre agreed with the members who thought there was no necessity for Dr. Choquet's paper, and that it did not fill a very obvious want. It seemed to him it was an opportune moment to urge on workers in the field of the bacteriology of dental caries the necessity of using standard media and of working out organisms according to some preconceived plan, and so adopting measures which would enable workers all over the world to identify the organisms and to realize whether those same organisms had been described before, and the names applied to them. In that way the list of organisms which were supposed to be concerned in the production of caries might be reduced very considerably, and those organisms might then be worked out more thoroughly. He was anxious to ask Dr. Choquet for some information about the various media with which he worked. In a paper in the *Dental Cosmos* a little while back, Dr. Choquet gave a list of twelve or fifteen media in which he grew his micro-organisms, and those media were so totally different from those generally used by bacteriologists that he might be describing organisms which were thoroughly well known and continually being found, but yet could not be identified by others on account of the peculiar methods by which Dr. Choquet prepared his media. Another point Dr. Choquet hardly seemed to realize was that when an organism was first isolated from the human body, that organism took a certain period of time—it might be a week, a fortnight, or two or three months—before it attained what might be called its "laboratory habit."

"An Attempt to explain the Sensitiveness of Dentine." By Dr. Alfred Gysi, Zürich.[1]

The author starts out with four fundamental propositions, in substance as follows:

1. No nerve-fibres have been found in the dentinal canals.

2. The dentinal canals are filled with a watery, organic substance, and exist in this form before a trace of nerve-fibre is to be demonstrated in the pulp. It is not probable that nerve-filaments grow from the pulp into the dentinal canals and displace the original contents.

3. At the inner border of the dentine about the odontoblasts a rich net-work of nerve-fibres exists.

4. From the science of physics it is known that water is prac-

[1] "Versuch zur Erklärung der Empfindlichkeit des Dentins." Von Dr. dent. surg. Alfred Gysi, Zürich. Schweizerische Vierteljahrsschrift für Zahnheilkunde, Januar, 1901.

tically incompressible, and when confined in an unyielding tube pressure exerted at one end of the tube is transferred without appreciable loss to the other end.

From the foregoing premises the author argues that a pressure or a pull upon the contents of the dentinal canals is immediately transmitted to the odontoblastic layer of the pulp, and so affects the nerves of the pulp and causes pain.

Pressure on the contents of dentinal canals is commonly produced by excavating instruments, and the reverse force by the action of salt, sugar, and other substances which have a great affinity for the watery contents of the dentinal tubules. In order to diminish the sensitiveness of dentine we must in some way impair the capacity of the contents of the tubules for transmitting hydrostatic impulses caused either by pressure or the reverse of pressure. When the contents of the tubules are dried in any way, they become less able to pass along impulses to the nerves of the pulp. Also, when the albumin of the watery contents is coagulated by chloride of zinc, carbolic acid, or other substances, the same result is obtained.

The author believes it to be impracticable to undertake the coagulation or modification of the contents of the dentinal tubule in a short time,—viz., five to fifteen minutes. Only a very slight depth can be reached in the time usually given to the treatment of sensitive dentine previous to excavating it. The way in which he would manage a sensitive cavity is as follows: trim away overhanging enamel edges, dry the cavity, and apply Salier's dental anæsthetic. Seal in the latter with zinc sulphate cement. The patient is then to be dismissed until another sitting, at which time the case is usually found quite manageable. I have been unable to find out the composition of "Salier's dental anæsthetic," although I have written to the author for information on the subject. It appears to be a secret preparation made in Germany and sold commonly in that country. Its action would seem to be the gradual coagulation of the albuminous contents of the tubules, thereby making them unable to convey force impulses into the region of the nerves of the pulp. The author gives the composition of his zinc sulphate cement as follows: The fluid: sulphate of zinc, 1 part by weight; water, 3 parts by weight. The powder consists of unglazed oxide of zinc. It is especially fitted for sealing in cavities, temporary treatments forming a sufficient stopping for a

*a*, impacted third molar in jaw of mummy; facet worn on molar.

short time, and being easily removed. The expense of the cement is very small, and can readily be compounded by an apothecary.

Appended to Dr. Gysi's article are eight histological plates, which are intended to establish the non-existence of nerve-filaments in the dentine, and to force me to the conclusion that the sensitiveness of dentine is due to the physical transmission of force by the contents of the tubuli to the odontoblastic layer of the pulp.

The author has given a very clear and useful exposition of the hydrostatic theory of the transmission of sensibility in the dentine, a theory which would seem to be a good working hypothesis, so long as the microscope fails to observe nerve-filaments. The adoption of this theory demands the use of sharp instruments in cutting dentine in order that all unnecessary force may be removed from the contents of the dentinal tubules, and it suggests a delicacy in the handling of cavities to which less accurate knowledge would probably be insensible.

The author's conclusion that a satisfactory modification of the sensitiveness of dentine cannot logically be expected in the few moments which the operator is accustomed to devote to it deserves careful consideration. The plan of applying medication and sealing it in the cavity for several days seems far more rational, and more consistent with the histological structure of the dentine.—William H. Potter.

## Figures and Tables

**Figure f1:**